# SUPPRESSOR OF MAX2 LIKE 6, 7, and 8 Interact with DDB1 BINDING WD REPEAT DOMAIN HYPERSENSITIVE TO ABA DEFICIENT 1 to Regulate the Drought Tolerance and Target *SUCROSE NONFERMENTING 1 RELATED PROTEIN KINASE 2.3* to Abscisic Acid Response in *Arabidopsis*

**DOI:** 10.3390/biom13091406

**Published:** 2023-09-18

**Authors:** Yuke Lian, Chengfei Lian, Lei Wang, Zhimin Li, Guoqiang Yuan, Lijuan Xuan, Huanhuan Gao, Haijun Wu, Tao Yang, Chongying Wang

**Affiliations:** Ministry of Education, Key Laboratory of Cell Activities and Stress Adaptations, School of Life Sciences, Lanzhou University, 222 Tianshui Road, Lanzhou 730000, China; lianyk18@lzu.edu.cn (Y.L.); lianchf20@lzu.edu.cn (C.L.); wanglei2020@lzu.edu.cn (L.W.); lizhm20@lzu.edu.cn (Z.L.); yuangq19@lzu.edu.cn (G.Y.); xuanlj17@lzu.edu.cn (L.X.); gaohh@lzu.edu.cn (H.G.); wuhj@lzu.edu.cn (H.W.)

**Keywords:** strigolactone, SMXL6,7,8, DWA1, drought tolerance, abscisic acid, *SnRKs*

## Abstract

SUPPRESSOR OF MAX2-LIKE 6, 7, and 8 (SMXL6,7,8) function as repressors and transcription factors of the strigolactone (SL) signaling pathway, playing an important role in the development and stress tolerance in *Arabidopsis thaliana*. However, the molecular mechanism by which SMXL6,7,8 negatively regulate drought tolerance and ABA response remains largely unexplored. In the present study, the interacting protein and downstream target genes of SMXL6,7,8 were investigated. Our results showed that the substrate receptor for the CUL4-based E3 ligase DDB1-BINDING WD-REPEAT DOMAIN (DWD) HYPERSENSITIVE TO ABA DEFICIENT 1 (ABA1) (DWA1) physically interacted with SMXL6,7,8. The degradation of SMXL6,7,8 proteins were partially dependent on DWA1. Disruption of SMXL6,7,8 resulted in increased drought tolerance and could restore the drought-sensitive phenotype of the *dwa1* mutant. In addition, SMXL6,7,8 could directly bind to the promoter of *SUCROSE NONFERMENTING 1 (SNF1)-RELATED PROTEIN KINASE 2.3* (*SnRK2.3*) to repress its transcription. The mutations in *SnRK2.2/2.3* significantly suppressed the hypersensitivity of *smxl6/7/8* to ABA-mediated inhibition of seed germination. Conclusively, SMXL6,7,8 interact with DWA1 to negatively regulate drought tolerance and target ABA-response genes. These data provide insights into drought tolerance and ABA response in *Arabidopsis* via the SMXL6,7,8-mediated SL signaling pathway.

## 1. Introduction

Drought is a potent abiotic stressor that hampers plant performance and induces yield loss by depressing their physio-biochemical and molecular functions [1]. Abscisic acid (ABA) is a main driver in response to a stressful environment that promotes stomatal closure and accelerates leaf senescence [2,3]. ABA is recognized by its receptors, PYRABACTIN RESISTANCE (PYR)/PYRABACTIN RESISTANCE-LIKE (PYL)/REGULATORY COMPONENT OF ABA RECEPTORS (RCARs), which inhibit TYPE 2C PROTEIN PHOSPHATASES (PP2Cs), releasing the inhibitory effect of PP2Cs on *SUCROSE NONFERMENTING 1 (SNF1)-RELATED PROTEIN KINASES* (SnRK2s) and allowing SnRK2s to activate downstream ABA-RESPONSIVE PROMOTER ELEMENTS (AREB)/ABA-BINDING FACTORS (ABFs) in ABA-responsive genes [4,5].

SnRK2s are a family of plant-specific protein kinases that positively regulate ABA signaling [6]. Among the 10 SnRKs (SNRK2.1-2.10) in *Arabidopsis*, SnRK2.2, SnRK2.3, and SnRK2.6 are functionally redundant and are critical for the subsequent response to abiotic stress in the ABA signaling pathway [7,8]. ABA activates the activity of SnRK2.2, SnRK2.3, and SnRK2.6 and promotes the degradation of SnRK2.3 through the 26S proteasome system [9]. The *snrk2.2/2.3/2.6* triple mutant exhibits more severe phenotypes in ABA signaling and water stress response compared with the wild type [10]. Overexpression of *TaSnRK2.3* significantly enhances drought tolerance in common wheat [11]. These studies suggest that the functions of SnRK2.2, SnRK2.3, and SnRK2.6 are essential for ABA signaling and drought stress tolerance. DDB1-binding WD-repeat domain (DWD) HYPERSENSITIVE TO ABA DEFICIENT 1 (ABA1) (DWA1) is a substrate receptor for the CUL4 (Cullin 4)-based E3 ligase and interacts with the ABSCISIC ACID INSENSITIVE 5 (ABI5) to accelerate its degradation to negatively regulate ABA signaling [12].

Strigolactones (SLs) are a class of carotenoid-derived plant hormones that positively regulate seed germination [13]; leaf senescence [14,15]; root architecture [16]; and plant responses to drought, salt [17], heat, and cold stresses [18]; but negatively regulate shoot branching [19,20,21] and the formation of adventitious and lateral roots [22]. SLs are perceived by their receptor DWARF 14 (D14), an α/β hydrolase that hydrolyses the SLs and then forms a complex with the F-box protein DWARF 3 (D3) in rice or MORE AXILLARY GROWTH 2 (MAX2) in *Arabidopsis* to recruit the repressors D53 in rice or SMXL6,7,8 in *Arabidopsis*, resulting in their degradation by the 26S proteasome [23,24].

SLs positively regulate drought tolerance [17,25,26]. Exogenous application of the synthetic SL analog *rac*-GR24 enhances the drought tolerance of wild-type plants [17]. SL-deficient *max3* and *max4* mutants, the SL-signaling *max2* mutant, and the SL receptor *d14* mutant are more susceptible to drought than the wild type in *Arabidopsis* [17,25,26]. Overexpression of *SsMAX2* of *Sapium sebiferum, MdMAX2*, and *MdD14* of *Malus domestica* (apple) in *Arabidopsis* improve drought and salt stress tolerance [27,28,29]. The transcriptional repressors SMXL6,7,8 negatively regulate drought tolerance in *Arabidopsis* [30,31,32]. SMXL6,7,8 act as repressors and can interact with other transcription factors to inhibit the expression of their downstream genes [21,23]. It has been reported that TaD53 interacts with SQUAMOSA PROMOTER BINDING PROTEIN-LIKE 3 (TaSPL3) and TaSPL17 in wheat; AtSPL9, AtSPL15, and AtBES1 in *Arabidopsis;* and BRASSINAZOLE RESISTANT 1 (OsBZR1) in rice to repress the expression of *TEOSINTE BRANCHED 1* (*TaTB1*), *BRANCHED1* (*BRC1*), and *FINE CULM 1* (*FC1*) and promote tillering/branching [33,34]. Moreover, D53 can also interact with IPA1 (OsSPL14) and represses IPA1-mediated *D53* expression in rice [35]. SMXL6,7,8 can directly bind to promoters of *SMXL6,7,8* to inhibit their transcription, suggesting that SMXL6,7,8 have a dual function in SL signaling [30,36].

ABA and SLs are two carotenoid-derived phytohormones that positively regulate drought tolerance in plants. ABA hypersensitivity and stronger drought tolerance were observed in the *smxl6/7/8* triple mutant, and drought-stress-inducible ABA-response genes were also up-regulated in *smxl6/7/8*, indicating that SMXL6,7,8 may participate in ABA-mediated drought response through transcriptional regulation of ABA response genes [31,32]. Chromatin immunoprecipitation sequencing (ChIP-seq) of SMXL6-HA had been reported, and many potential downstream target genes of SMXL6 were found by Wang et al. [30]. These data led us to ask whether SMXL6,7,8, as transcription factors, participate in drought stress and ABA response through binding to downstream target genes. However, the underlying mechanism is not completely clear, and it is challenging to explore a new regulatory pathway that contributes to drought tolerance and ABA response in plants. Are SMXL6,7,8 involved in the regulation of drought tolerance by interacting with other proteins? Do SMXL6,7,8, as transcription factors, regulate drought tolerance and ABA response by inhibiting the transcription of ABA/drought response genes? These issues are not clear.

In this study, we screened a protein (DWA1) interacting with SMXL6,7,8 that promoted the degradation of SMXL6,7,8 in a 26S proteasome-dependent pathway. Furthermore, SMXL6,7,8 directly bound to the promoter of *SnRK2.3* to repress its transcription and then its response to ABA-mediated seed germination. Accordingly, this work further reveals a possible molecular mechanism by which SMXL6,7,8 regulate drought tolerance and ABA response in plants.

## 2. Materials and Methods

### 2.1. Plant Materials and Growth Conditions

The wild-type *Arabidopsis thaliana* ecotype Columbia (Col-0) was used. The T-DNA insertion mutants *smxl6* (SAIL_1285_H05), *smxl7* (SALK_082032), *smxl8* (SALK_126406), *dwa1-1* (SALK_051022), and *dwa1-2* (SALK_021789) were obtained from the Nottingham *Arabidopsis* Stock Centre (NASC, Nottingham, UK, http://arabidopsis.info/ (accessed on 7 August 2019)). These seeds were used in previous studies [12,21,37]. Seeds of the *snrk2.2/2.3* mutant in the Col-0 background were originally generated by Hiroaki et al. (2007) [7]. The multiple mutants, including *smxl6/7/8*, *smxl6/7/8/dwa1-1*, *smxl6/7/8/dwa1-2*, *smxl6/7/8/snrk2.3*, and *smxl6/7/8/snrk2.2/2.3*, and the single mutant *snrk2.3* were generated via genetic crossing in this study, and homozygous lines were identified and used for genetic analyses. The genotyping primers are described in Table S1. Seeds were germinated on ½ MS medium with 1% sucrose and 1% agar after sterilization (75% ethanol for 45 s and then with 0.1% HgCl_2_ for 5 min, then rinsed five times in sterilized water). Seven-day-old seedlings were transplanted into the soil in a standard growth condition with a 16-hour-light/8-hour-dark photoperiod and 60% relative humidity at 22 °C.

### 2.2. Seed Germination Assay

All the seeds of Col-0, *snrk2.3*, *snrk2.2/2.3*, *smxl6/7/8*, *smxl6/7/8snrk2.3*, and *smxl6/7/8/snrk2.2/2.3* were germinated and grown on ½ MS medium with or without 0.5 μM and 1 μM ABA (Solarbio, Beijing, China), respectively. The percentage of germinated seeds was recorded every day, and the phenotypes were photographed at 6 days after sowing.

### 2.3. Drought Stress and Relative Water Content (RWC) Analysis

Drought stress was induced with modification [31]. In brief, seedlings were grown on ½ MS medium (containing 1% sucrose and 1% agar) for 7 days under a 16-hour-light/8-hour-dark photoperiod at 22 °C. A total of 16 seedlings were transplanted into a small pot containing the same weight of soil, and these continued to grow for 3 weeks. Then, water was withheld from the plants until a phenotype appeared and was photographed. The plants were photographed again after 3 days of rewatering. The survival rates of multiple drought experiments were calculated. For relative water content (RWC) analysis, 30 rosette leaves from 4-week-old plants were detached and weighed at the indicated times [17]. The experiment was performed with at least three biological replicates and at least three times with similar results.

### 2.4. Yeast Two-Hybrid (Y2H) and Yeast One-Hybrid (Y1H) Assays

To generate constructs fused to the GAL4 DNA-binding domain (BD) and activation domain (AD), the full-length coding sequences were cloned into the *EcoR*I-*BamH*I sites of the *pGBKT7* and *pGADT7* vectors, respectively. The primers and cloning sites used for plasmid construction are given in Table S1. The yeast assays were performed as described previously with modifications [38]. The yeast transformation and two-hybrid library screen were performed according to the manufacturer’s instructions (Clontech, Mountain View, CA, USA, https://www.takarabio.com/ (accessed on 5 November 2018)). Reagents used in yeast experiments were from Clontech and Solarbio. The cDNA library was from Clontech (Make Your Own “Mate & Plate” Library System, Code No. 630490). The Y2H library was screened via the yeast-mating method. Briefly, *pGBKT7-SMXL6*, *pGBKT7-SMXL7*, and *pGBKT7-SMXL8* constructs were transformed into Y2H Gold as bait. The library strains were combined with bait strains after autoactivation and toxicity tests. Subsequently, the candidate interacting proteins of SMXL6, SMXL7, and SMXL8 were screened using yeast two-hybrid libraries, and then all positive clones were sequenced.

For the Y2H assays, bait and prey constructs were co-transformed into Y2H Gold and spotted onto plates containing double-dropout (DDO) medium without leucine and tryptophan (SD/-Leu/-Trp) or quadruple-dropout (QDO) medium without leucine, tryptophan, adenine, and histidine (SD/-Ade/-His/-Leu/-Trp) with or without X-α-Gal (5-Bromo-4-chloro-3-indoxyl-α-D-galactopyranoside)/Aureobasidin A (AbA, a cyclic depsipeptide antifungal antibiotic) and incubated at 30 °C for 3 days.

For the Y1H assays, the yeast strain EGY48 was used for the co-transformation of plasmids for *pGADT7-SMXL6*,*7*,*8* and the *LacZ* reporters driven by various promoter fragments. Their binding was observed according to X-Gal (5-bromo-4-chloro-3-indolyl-β-D-galactopyranoside) chromogenic reaction on medium (SD/-Leu/-Ura) agar plates after 3 days at 30 °C.

### 2.5. GST Pull-Down Assay

A GST pull-down assay was conducted with modification [38]. The coding regions of *SMXL6*, *7*, and *8* were cloned into *pGEX-6P-1*, and *DWA1* was cloned into *pET-30a* to obtain the GST-SMXL6, GST-SMXL7, GST-SMXL8, and His-DWA1 recombinant proteins, respectively. The plasmids were grown in *E. coli*. After ultrasonication, GST-SMXL6, GST-SMXL7, GST-SMXL8, and GST supernatant as bait were incubated with Beaver Beads™ GSH (BEAVER) in PBS (140 mM NaCl, 2.7 mM KCl, 20 mM Na_2_HPO_4_, and 1.8 mM NaH_2_PO_4_), 1% Triton X-100, 1 mM PMSF, and 1% BSA for 4 h at 4 °C. After the Beaver Beads™ GSH were washed 3 times for 5 min with buffer (1× PBS, 1% Triton X-100, and 2 mM DTT), the extract from cells producing His-DWA1 was added as prey and incubated for a further 1 h. The beads were washed ten times with wash buffer (1× PBS, 1% Triton X-100, 2 mM DTT). The input and proteins retained on the beads were separated on 12% and 8% SDS-PAGE gels and then analyzed via immunoblotting with anti-His monoclonal antibody (1:5000; Abmart, Shanghai, China, Catalog No. M20001) or anti-GST monoclonal antibody (1:5000; Abmart, Catalog No. M20007).

### 2.6. Bimolecular Fluorescence Complementation (BiFC) Assay

The coding regions of *SMXL6,7,8* and *DWA1* were cloned next to the N-terminal part of YFP (*nYFP*) in the *pEarleygate202-YN* vector and the C-terminal part of YFP (*cYFP*) in the *pEarleygate201-YC* vector. Different constructs (including empty vectors) were transformed into the Agrobacterium strain GV3101 and then infiltrated into *Nicotiana benthamiana* leaves for transient expression. Fluorescence was observed and photographed in leaf epidermal cells 2 days after infiltration using a Confocal laser scanning microscope (Nikon, A1R+Ti2-E, Tokyo, Japan).

### 2.7. Cell-Free Degradation Assay

Cell-free assays were conducted with modification [12]. Briefly, 10-day-old seedlings of wild-type and *dwa1-1* mutants growing on ½ MS were extracted in a buffer (25 mM Tris, pH 7.5, 10 mM MgCl_2_, 5 mM DTT, and 10 mM NaCl). Purified GST-SMXL6, GST-SMXL7, and GST-SMXL8 fusions (1 μg) were mixed with cell extracts (10 μg) with 10 mM ATP and incubated at 30 °C for 1 h with or without 50 mM MG132 (carbobenzoxyl-L-leucyl-L-leucyl-L-leucine, a proteasome-specific inhibitor). Equal amounts of samples were separated by 8% SDS-PAGE gels and analyzed via immunoblotting with an anti-GST antibody.

### 2.8. Constructs for Overexpression and Green Fluorescent Protein (GFP) Fluorescence Assay

To generate the *35S::SMXL6,7,8-GFP* (*OE-SMXL6,7,8*) construct, the full-length coding sequences of *SMXL6,7,8* were individually cloned into the *Kpn*I-*Sal*I sites of the *pCAMBIA1300-GFP* vector via the in-fusion method (Clontech). The primers are shown in Table S1. To generate *OE-SMXL6*, *OE-SMXL7*, and *OE-SMXL8* transgenic plants, the corresponding constructs were introduced into the *Agrobacterium* strain GV3101 and then into the wild type via the floral dip method [39]. Homozygous *OE-SMXL6-4*, *OE-SMXL7-1*, and *OE-SMXL8-2* were introduced into *dwa1-1* to obtain *OE-SMXL6/dwa1-1*, *OE-SMXL7/dwa1-1*, and *OE-SMXL8/dwa1-1* lines via genetic crossing, respectively.

For the fluorescence observation of the *OE-SMXL6/7/8* and *OE-SMXL6/7/8/dwa1* transgenic lines, these seeds were sown in the same ½ MS medium plate and subjected to a 16-hour-light/8-hour-dark photoperiod and 60% relative humidity at 22 °C in a greenhouse. Fluorescence observation was performed after 5 days. The seedlings of two transgenic lines, such as *OE-SMXL6* and *OE-SMXL6/dwa1*, were placed under the same slide for observing using the layer scanning method with a confocal laser scanning microscope (Nikon, A1R + Ti2-E, Tokyo, Japan). Then, multiple-layer fluorescent images were superimposed into a single image using a stacking technique in Photoshop. Finally, the overall fluorescence in the multiple roots of the two lines were counted.

### 2.9. Toluidine Blue Staining

Toluidine blue (TB) staining was conducted as described previously with modification [40]. The fifth leaves of 4-week-old plants were detached, immersed into a solution of 0.05% (*w*/*v*) TB, and softly shaken for 3 h. The excessive TB was washed with water, and then the leaves were photographed.

### 2.10. Immunoblot Analysis

The seeds of Col-0, *OE-SMXL6*, *OE-SMXL7*, and *OE-SMXL8* lines were grown on ½ MS for 7 days, and their seedlings were harvested and extracted in a buffer (1 M Tris-MES, pH 8.0, 2 M sucrose, 1 mM MgCl_2_, 0.5 M EDTA, 2 M DTT, and 0.1 M PMSF). The samples with equal amounts were separated by 8% SDS-PAGE gels and analyzed via immunoblotting with an anti-GFP antibody (1:5000; Abmart, Catalog No. M20004M). The Anti-Actin antibody (1:5000; Abmart, Catalog No. M20009S) was used as a normalization control.

### 2.11. Quantitative Real-Time PCR (qRT-PCR) Assay

qRT-PCR was performed as described previously with modifications [31]. Briefly, the total RNA of 10-day-old wild type, *smxl6/7/8*, *OE-SMXL6*, *OE-SMXL7*, and *OE-SMXL8* seedlings were prepared using a MiniBEST Plant RNA Extraction Kit (Takara, Beijing, China) according to the users’ manual. RNA samples were reverse-transcribed using a PrimeScript^TM^ II 1st Strand cDNA Synthesis Kit (Takara). The qRT-PCR experiments were finished using gene-specific primers (Table S1) on a real-time system (ABI Q5) in a total volume of 20 μL containing 8 μL diluted cDNA, 0.5 μM gene-specific primers, and 10 μL ChamQ Universal SYBR qPCR Master Mix (Vazyme, Nanjing, China). The Arabidopsis *ACTIN2* gene was used as the internal control. All experiments were set up with three biological replicates and three technical replicates.

### 2.12. Transient Expression Assays and Dual-Luciferase Reporter Assay

Transient expression in the *Arabidopsis* mesophyll cells was performed accordingly [41]. The coding region of *SMXL6*, *SMXL7*, and *SMXL8* were each cloned to *pGreenII 62-SK* vectors containing the 35S promoter. The *pGreenII 62-SK-GFP* was used as a control. To generate the *pGreenII0800-LUC-SnRK2.3/2.6*, the 2000 bp sequence upstream of the ATG start codon was amplified via PCR using genomic DNA as the template and the pair of primers (Table S1).

The dual-luciferase reporter assay was performed as described previously [30]. *pGreenII 62-SK-GFP* or *pGreenII 62-SK-SMXL6*,*7*,*8* were co-transformed into *Arabidopsis* protoplasts with *pGreenII 0800-LUC-SnRK2.3/2.6*, respectively. After overnight incubation in the dark, the protoplasts were centrifuged. The transient expression vector *pGreenII 0800-LUC* construct used in our experiment contains a *REN* gene controlled by a 35S promoter to provide an estimate of the extent of transient expression. The Renilla luciferase gene (*REN*) was used as an internal control. A Dual-Luciferase Reporter Assay Kit (Promega, Madison, WI, USA) was used to detect firefly luciferase (LUC) activity and the Renilla luciferase gene (*REN*) using a GloMax^®^ 20/20 Luminometer (Promega). The relative luciferase activity was expressed as Firefly Luc/Renilla Luc.

### 2.13. Electrophoretic Mobility Shift Assay (EMSA)

EMSAs were performed as described previously with minor modifications [30]. The sequences of the biotin-labeled probes are listed in Table S1. In brief, 1 μg of purified GST or GST-SMXL6,7,8 proteins were incubated together with biotin-labeled probes in 20 μL reaction mixtures, with purified GST protein used as a competitive cold probe. The binding reactions were allowed to proceed at room temperature for 30 min and were halted by adding the 5 μL 5× loading buffer, and then they were separated with 6% native polyacrylamide gel in pre-cooled 0.5× TBE (Tris-base, Boric Acid, EDTA) buffer.

### 2.14. Statistical Analysis

Three independent experiments were performed, and results were expressed as the mean ± the standard deviation (SD). Data were analyzed by using GraphPad Prism 7 software. Statistical analyses were performed in SPSS Statistics (IBM, Tulsa, OK, USA). We used a one-way analysis of variance (ANOVA) with Duncan’s multiple range tests and a Student’s *t*-test.

## 3. Results

### 3.1. SMXL6,7,8 Proteins Physically Interact with the DWA1 Protein

**Figure 1:**
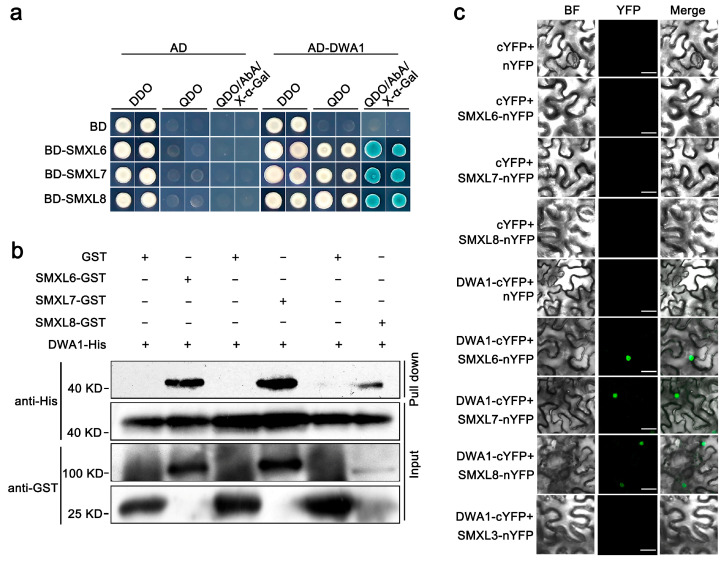
SMXL6,7,8 physically interact with DWA1. (**a**) Yeast two-hybrid assays of the interactions between SMXL6,7,8 and DWA1. The full-length SMXL6,7,8 proteins were fused to the GAL4 binding domain (BD), and the DWA1 protein was fused to the GAL4 activation domain (AD). DDO: SD/-Leu/-Trp; QDO: SD/–Ade/–His/–Leu/–Trp; X-α-Gal: 5-Bromo-4-chloro-3-indoxyl-α-D-galactopyranoside; AbA: Aureobasidin A (a cyclic, depsipeptide antifungal antibiotic). (**b**) Pull-down assay for detecting the interaction between GST-SMXL6,7,8 and His-DWA1. GST, GST-SMXL6,7,8, and His-DWA1 were expressed in the *Rosetta (DE3) E. coli* strain. Purified proteins were used for the pull-down assay. GST and GST-SMXL6,7,8 were bound to GST beads as baits and His-DWA1 was used as prey. The His-DWA1 protein was detected with anti-His antibodies, and the GST and GST-SMXL6,7,8 proteins were detected with anti-GST antibodies. The GST protein was used as a control. “+” indicates that a relative protein has been added in lane, “−” indicates that no protein has been added. (**c**) Bimolecular fluorescence (BiFC) assay of the interactions between SMXL6,7,8 and DWA1. *N. benthamiana* leaves co-expressing SMXL6,7,8-nYFP and DWA1-cYFP fusion proteins were observed 2 days after infiltration. Co-expression of SMXL6,7,8-nYFP and cYFP, nYFP, and DWA1-cYFP or SMXL3-nYFP and DWA1-cYFP served as negative controls. The YFP signals were observed using confocal microscopy, and green pseudo-colour was used to indicate the YFP fluorescence signal generated by the interaction of the two proteins. Scale bars = 20 μm. The experiments were repeated three times with similar results.

To further uncover the molecular mechanism of SMXL6,7,8 regulating drought stress, a normalized *Arabidopsis* Y2H cDNA library was used as the prey to identify the candidate interactors of SMXL6,7,8. The full-length *SMXL6,7,8* sequences were individually fused to the GAL4 DNA binding domain of the bait vector (BD-SMXL6, BD-SMXL7, and BD-SMXL8). We screened out the interacting clone *DWA1*, which encodes a substrate receptor for CUL4 E3 ligases. To confirm the interaction between SMXL6,7,8 and DWA1 in yeast, we introduced the full-length *DWA1* into the GAL4 activation domain of the prey vector (AD-DWA1). The BD-SMXL6,7,8 and AD-DWA1 plasmids were co-transformed into yeast. The Y2H assays showed that SMXL6,7,8 interacted with DWA1 in yeast (Figure 1a). Furthermore, a GST-pull down assay was performed with recombinant GST-SMXL6, GST-SMXL7, GST-SMXL8, and His-DWA1 proteins, which showed that SMXL6,7,8 could directly interact with DWA1 in vitro (Figure 1b). The physical interaction between SMXL6,7,8 and DWA1 was further confirmed by a BiFC analysis. SMXL6, SMXL7, and SMXL8 were separately fused to the N-terminal yellow fluorescent protein (YFP) fragment (SMXL6,7,8-nYFP), and DWA1 was ligated with the C-terminal YFP fragment to generate DWA1-cYFP. When SMXL6-nYFP, SMXL7-nYFP, and SMXL8-nYFP were co-infiltrated with DWA1-cYFP into *N. benthamiana* leaves, the YFP signal was localized in the nucleus, while no signal was detected in negative controls in which SMXL6,7,8-nYFP were individually co-expressed with cYFP or DWA1-cYFP was co-expressed with nYFP (Figure 1c). The SMXL family protein SMXL3 also did not interact with DWA1 in BiFC (Figure 1c). Together, these data provide evidence that SMXL6,7,8 physically interact with DWA1 in vitro and in vivo.

### 3.2. The Degradation of SMXL6,7,8 Proteins Partially Depend on DWA1

**Figure 2:**
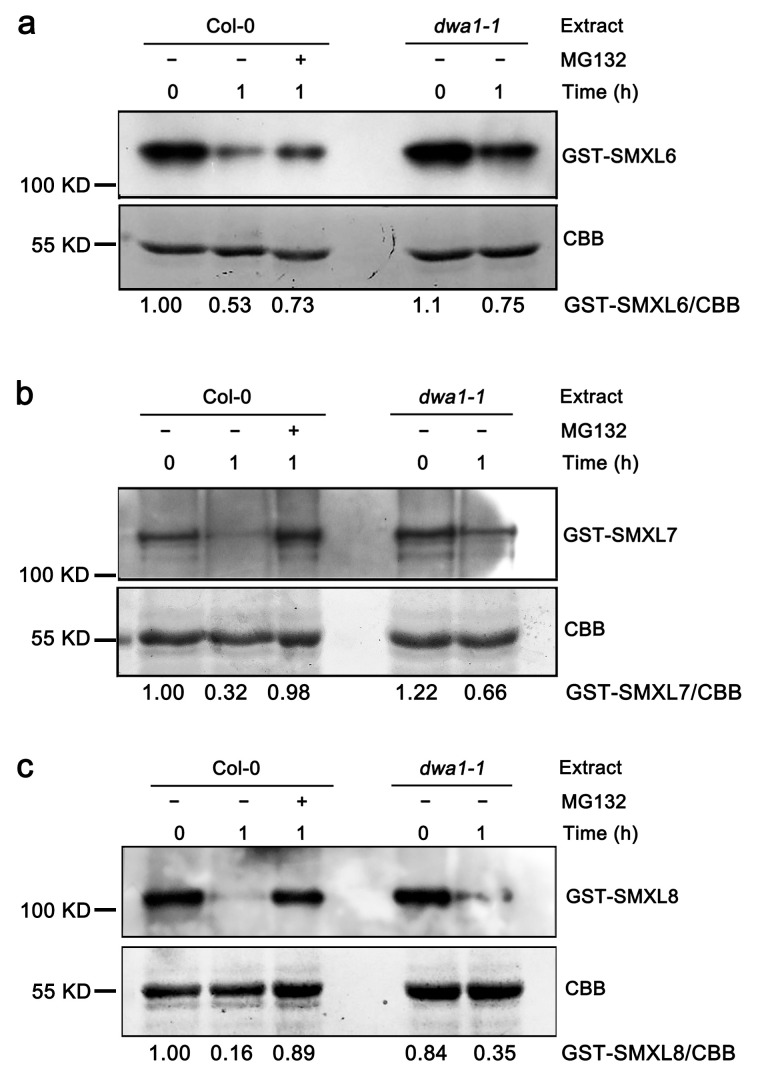
DWA1 partially promotes the degradation of SMXL6,7,8 in vitro. (**a**–**c**) Degradation of GST-tagged SMXL6 (**a**), SMXL7 (**b**), and SMXL8 (**c**) proteins in wild-type and *dwa1-1* extracts. GST-SMXL6,7,8 proteins (1 μg) were incubated with distinct extracts (10 μg) prepared from 10-day-old seedlings (grown in ½ MS) with or without 50 μM MG132 for 1 h at 30 °C. GST-SMXL6,7,8 levels were examined via immunoblotting with anti-GST antibody. Coomassie brilliant blue-stained Rubisco (CBB) was used as a loading control.

DWA1 acts as the substrate receptor of the CUL4-based E3 ligase complex, which functions in ubiquitination [12]. Therefore, we tested whether the stability of the SMXL6,7,8 proteins were affected by DWA1. GST-tagged SMXL6,7,8 recombinant proteins were incubated with protein extracts prepared from the Col-0 and *dwa1-1* in the presence of ATP and analyzed via immunoblotting. Cell-free degradation assays showed that the recombinant GST-SMXL6, GST-SMXL7, and GST-SMXL8 proteins were degraded in the wild-type extracts, and their degradation was partially blocked by MG132, a proteasome-specific inhibitor (Figure 2). However, the GST-SMXL6, GST-SMXL7, and GST-SMXL8 proteins were degraded more slowly in *dwa1-1* than in the wild-type extracts (Figure 2), suggesting that the degradations of SMXL6,7,8 are partially dependent on DWA1.

**Figure 3:**
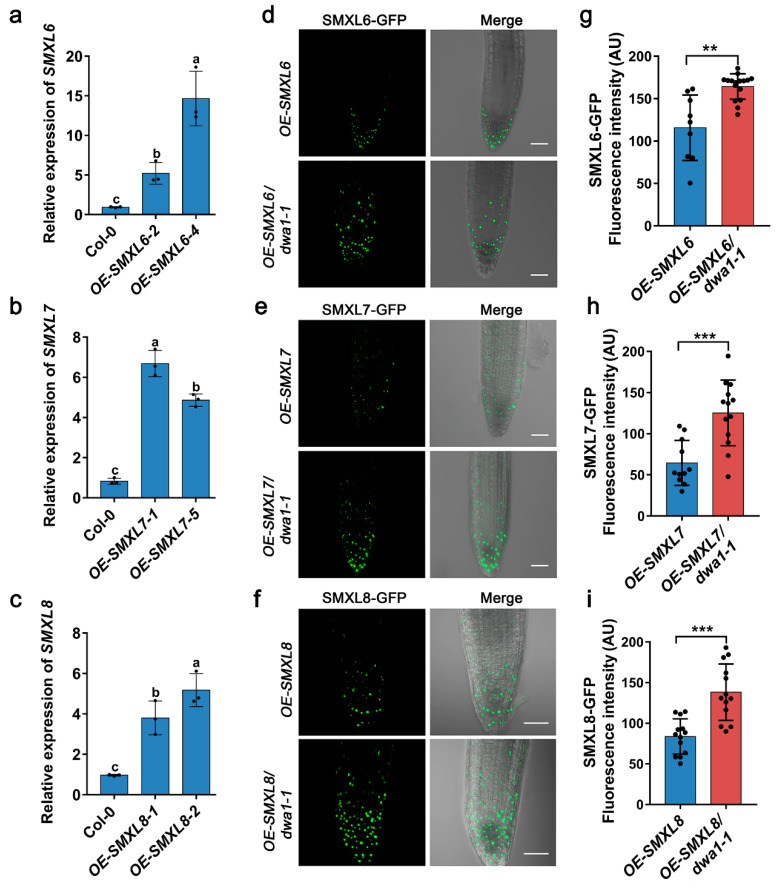
The accumulation of SMXL6,7,8 proteins in root tips of the wild type and *dwa1-1*. (**a**–**c**) The relative expression of *SMXL6*, *SMXL7*, and *SMXL8* in transgenic lines, respectively. *UBQ10* was used as the reference gene. The data are mean values ± SD of three replicates. Different letters represent statistical differences at *p* < 0.05 (according to the Duncan’s multiple range test). (**d**–**f**) Observation of SMXL6,7,8-GFP fluorescence signals in the wild type and *dwa1-1*. *35S::SMXL6-GFP (OE-SMXL6-4), 35S::SMXL7-GFP (OE-SMXL7-1)*, and *35S::SMXL8-GFP (OE-SMXL8-2)* lines in the wild type were genetically crossed with the *dwa1-1* mutant to generate the *OE-SMXL6/dwa1-1, OE-SMXL7/dwa1-1*, and *OE-SMXL8/dwa1-1* lines, respectively. All GFP fluorescence signals in root tips of 5-day-old seedlings were observed via the layer scanning method using a confocal laser scanning microscope. (**g**–**i**) The measurement of GFP fluorescence intensity of *OE-SMXL6,7,8* and OE-*SMXL6,7,8/dwa1-1.* The fluorescence intensity was counted by using Image J. The data points represent the overall fluorescence from an individual root of each line. Scale bars = 50 μm. Data are means ± SD (*n* ≥ 10). Asterisks indicate a significant difference (** *p* < 0.01, *** *p* < 0.001) as determined by a Student’s *t*-test.

To further verify these data, we first generated *OE-SMXL6*, *OE-SMXL7*, and *OE-SMXL8* transgenic plants in a wild-type background, and the expressions of *SMXL6,7,8* were verified with a qRT-PCR assay (Figure 3a–c). The results of the Western blot showed that the levels of SMXL6, SMXL7, and SMXL8 proteins were higher in the *OE-SMXL6-4*, *OE-SMXL7-1*, and *OE-SMXL8-2* lines, respectively (Figure S1), which was consistent with the results of the qRT-PCR. Next, *OE-SMXL6-4*, *OE-SMXL7-1*, and *OE-SMXL8-2* lines with higher expression levels were introduced into the *dwa1-1* background via genetic crossing. Then, the SMXL6-GFP, SMXL7-GFP, and SMXL8-GFP fluorescence of the root tips were observed. The results showed that the fluorescence intensity in the *OE-SMXL6/dwa1-1*, *OE-SMXL7/dwa1-1*, and *OE-SMXL8/dwa1-1* plants was higher than in the *OE-SMXL6*, *OE-SMXL7*, and *OE-SMXL8* plants (Figure 3d–i), suggesting that the degradations of SMXL6,7,8 are at least partially dependent on the DWA1 protein.

### 3.3. SMXL6, 7, and 8 Are Epistatic to DWA1 in Response to Drought Tolerance

**Figure 4:**
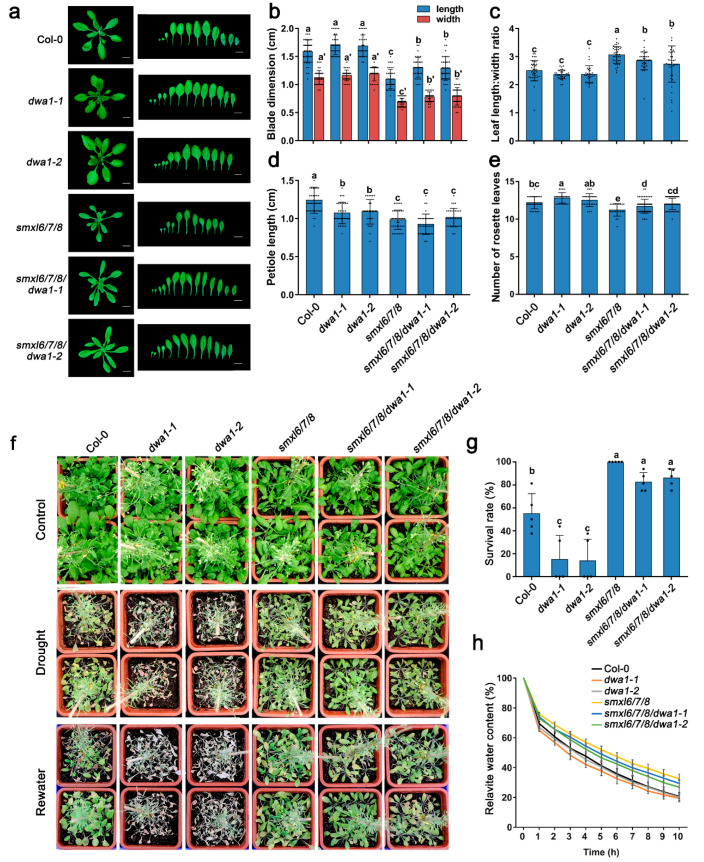
The leaf morphology and drought tolerance phenotypes of the wild type (Col-0), *dwa1-1*, *dwa1-2*, *smxl6/7/8*, *smxl6/7/8/dwa1-1*, and *smxl6/7/8/dwa1-2.* (**a**) Representative images and rosette leaves from 35-day-old plants of wild-type Col-0, *dwa1-1*, *dwa1-2*, *smxl6/7/8*, *smxl6/7/8/dwa1-1*, and *smxl6/7/8/dwa1-2.* Scale bars = 1 cm. (**b**–**e**) The blade length (black, not including the petiole) and width (gray) (**b**), leaf length:width ratio (**c**), petiole length (**d**), and number of primary rosette leaves (**e**) were determined for the 7th–8th leaf of 26-day-old plants of the wild type, *dwa1-1*, *dwa1-2*, *smxl6/7/8*, *smxl6/7/8/dwa1-1*, and *smxl6/7/8/dwa1-2* grown under an LD photoperiod (16 h light/8 h dark). Data are mean values ± SD (*n* = 35–40). (**f**) Drought tolerance assays of the wild-type, *dwa1-1*, *dwa1-2*, *smxl6/7/8*, *smxl6/7/8/dwa1-1*, and *smxl6/7/8/dwa1-2* lines. Three-week-old plants in the soil were subjected to drought stress by withholding water for 16 days. The plants were re-watered for 3 days. (**g**) The survival rates of the wild type, *dwa1-1*, *dwa1-2*, *smxl6/7/8*, *smxl6/7/8/dwa1-1*, and *smxl6/7/8/dwa1-2* after re-watering. Data are the mean ± SD of the survival rates of five groups (16 plants per group) in one experiment. (**h**) Relative water content in the wild type, *dwa1-1*, *dwa1-2*, *smxl6/7/8*, *smxl6/7/8/dwa1-1*, and *smxl6/7/8/dwa1-2*. Four-week-old plants were used for assaying the water content in detached leaves. The experiment was performed with three biological replicates and was repeated three times with similar results. The data are mean values ± SD of three replicates. Different letters represent statistical differences at *p* < 0.05 (according to Duncan’s multiple range test).

To further explore the genetic interactions between SMXL6,7,8 and DWA1, we generated the quadruple mutants *smxl6/7/8/dwa1-1* and *smxl6/7/8/dwa1-2* by crossing two T-DNA insertion mutants (*dwa1-1* and *dwa1-2*) with *smxl6/7/8* triple mutant plants. The *dwa1-1* and *dwa1-2* T-DNA insertions disrupted the production of full-length transcripts (Figure S2a,b). The two *dwa1* mutants had slightly shortened petiole lengths and increased numbers of rosette leaves. Also, *smxl6/7/8* showed several phenotypes, including a reduced blade dimension, an increased leaf length–width ratio, a shortened petiole length, and reduced numbers of rosette leaves compared with the wild-type Col-0, while the *smxl6/7/8/dwa1-1* and *smxl6/7/8/dwa1-2* mutants had an enlarged blade dimension, a shortened petiole length, a decreased leaf length–width ratio, and increased numbers of rosette leaves compared with *smxl6/7/8* (Figure 4a–e).

We then investigated whether SMXL6,7,8 and DWA1 function together in response to drought stress. Under drought stress conditions, the *dwa1-1* and *dwa1-2* plants showed drought sensitivity compared with the wild type (Figure 4f, Figures S2c and S3), while the *smxl6/7/8* triple mutant plants displayed an enhanced drought tolerance (Figure 4f and Figure S3). *dwa1* had a lower survival rate and *smxl6/7/8* had a higher survival rate than the wild type when deprived of water for 16 days (Figure 4g and Figure S3). Simultaneously, the *OE-SMXL6,7,8* lines were sensitive to drought stress (Figure S4). This proved that DWA1 positively regulates plant drought tolerance, while SMXL6,7,8 negatively regulates plant drought tolerance. The drought tolerance of two *smxl6/7/8/dwa1* quadruple mutants was similar to that of the *smxl6/7/8* triple mutant plants when deprived of water for 16 days (Figure 4f and Figure S3), while the drought tolerance of *smxl6/7/8/dwa1-1* was slightly weaker than that of *smxl6/7/8* when deprived of water for 18 days (Figure S3). In addition, the relative water content of the *dwa1-1* mutant was lower than that of the wild type. In contrast, the relative water content of the *smxl6/7/8* mutant was higher than that of the wild type, and one of the *smxl6/7/8/dwa1* quadruple mutants was lower than that of *smxl6/7/8* (Figure 4h). It is well known that the permeability of the cuticle is also an important indicator of drought susceptibility [42]. TB staining was used to visualize the potential differences of cuticle permeability among the lines described above. Compared with that of the wild type, the TB staining of *dwa1* was enhanced, while that of the *smxl6/7/8* triple mutant was reduced, and that of the *smxl6/7/8/dwa1* mutants was similar to that of *smxl6/7/8*, which was consistent with the drought phenotype (Figure S5). Taken together, these findings indicate that SMXL6,7,8 act downstream of DWA1 to negatively regulate drought tolerance.

### 3.4. SMXL6,7,8 Directly Bind to the Promoter of SnRK2.3

A recent study reported that SMXL6,7,8 function as transcription factors by binding to the promoters and inhibiting the transcription of genes encoding downstream transcription factors. Deep-clustering GO enrichment analysis of previously published chromatin immunoprecipitation sequencing (ChIP-seq) data revealed 729 genes targeted by SMXL6-HA [30], and the results showed that SMXL6-regulated candidate genes were enriched for the pathways such as ‘response to the ABA’, ‘response to water deprivation’, and ‘response to osmotic stress’ (Figure S6a). Here, we selected some interesting genes in ‘response to the ABA’ to verify whether they could function as target genes of SMXL6,7,8 (Figure S6b). We then found that *SnRK2.3*, a member of ABA signaling, may be a candidate target gene for SMXL6 (Figure S6b).

**Figure 5:**
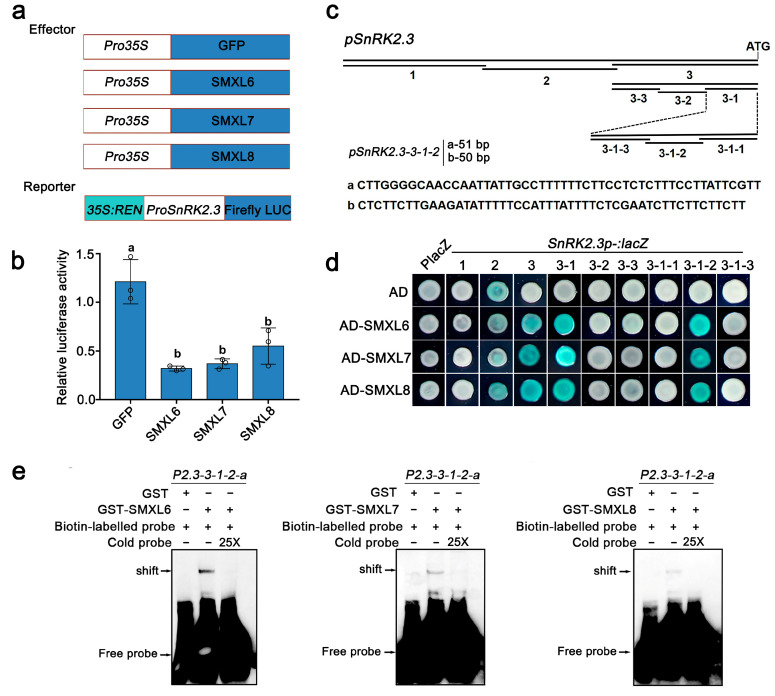
SMXL6,7,8 directly bind to the *SnRK2.3* promoter. (**a**) Schematic diagram of dual-luciferase assay constructs. The promoter of *SnRK2.3* was fused to the *REN* gene controlled by a *35S* promoter and *LUC* gene as a reporter, and the effector constructs contained a *GFP*, *SMXL6*, *SMXL7*, or *SMXL8* gene driven by the CaMV 35S promoter. (**b**) Transcriptional activities of SMXL6,7,8 on the *SnRK2.3* promoter in *Arabidopsis* protoplasts. The experiment was independently repeated three times with three biological replicates in each experiment, and similar results were obtained. Data are the means of three biological replicates ± SD. Different letters represent statistical significance at *p* < 0.05 (according to Duncan’s multiple range test). (**c**) Truncated regions of the *SnRK2.3* promoter and synthesized fragments of EMSA probes. (**d**) Yeast one-hybrid assays showing that SMXL6,7,8 displayed strong binding to the fragment 3-1-2 of the *SnRK2.3* promoter. An empty vector expressing the AD domain alone was used as the negative control. (**e**) EMSAs showing that the SMXL6,7,8 proteins specifically bound to fragments ‘3-1-2-a’ of the *SnRK2.3* promoter in vitro. For the competition, 25× excess unlabeled probes (cold probes) were mixed with biotin-labeled probes. GST, glutathione-S-transferase. Data represent three independent experiments.

SnRK2.3 is a central component of ABA signaling and is functionally redundant with SnRK2.2 and SnRK2.6 in the positive regulation of drought stress [7,8,43]. As SnRK2.3 and SMXL6,7,8 display opposite regulatory effects on the ABA response and drought stress [31,44,45], we investigated if SMXL6,7,8 inhibit the transcription of *SnRK2.3* using a dual-luciferase reporter assay in *Arabidopsis* mesophyll protoplasts. As expected, the overexpression of individual SMXL6, SMXL7, and SMXL8 inhibited the activity of LUC driven by the *SnRK2.3* promoter compared with the expression of GFP alone (Figure 5a,b). Next, we further performed Y1H assays to investigate whether SMXL6,7,8 could directly bind to the *SnRK2.3* promoter. The results of multiple rounds of promoter truncation showed that a 101 bp fragment (fragment p2.3-3-1-2) upstream of the start codon of the *SnRK2.3* gene was sufficient for SMXL6,7,8 to bind to *SnRK2.3* (Figure 5c,d). Then, we truncated ‘p2.3-3-1-2’ into the two segments ‘p2.3-3-1-2-a’ and ‘p2.3-3-1-2-b’ and validated the binding of SMXL6,7,8 to the *SnRK2.3* promoter via EMSA, in which the results showed that GST-SMXL6, 7, and 8 could directly bind to the probes of the ‘p2.3-3-1-2-a’ and ‘p2.3-3-1-2-b’ segments of *SnRK2.3*, respectively (Figure 5c and Figure S7). Together, our data demonstrate that SMXL6, 7, and 8 may inhibit the transcription of *SnRK2.3* by directly binding to its promoter.

Given the functional redundancy between *SnRK2.3*, *SnRK2.2*, and *SnRK2.6*, we also detected the binding of SMXL6,7,8 to the *SnRK2.2* and *SnRK2.6* promoters via Y1H. Similar to *SnRK2.3*, the overexpression of individual SMXL6, SMXL7, and SMXL8 also inhibited the activity of LUC driven by the *SnRK2.6* promoter compared with the expression of GFP alone (Figure S8a,b). Multiple rounds of promoter truncation showed that a 112 bp fragment (fragment p2.6-3-1-1) upstream of the start codon of the *SnRK2.6* gene was sufficient for SMXL6,7,8 to bind to *SnRK2.6* (Figure S8c,d). The EMSA results showed that SMXL6 and SMXL8 could bind to the ‘p2.6-3-1-1-a’ segment (Figure S8). However, we found that SMXL6,7,8 could not bind to the promoter 2000 bp upstream of the *SnRK2.2* ATG (Figure S9). The results of the qRT-PCR showed that the relative expression of *SnRK2.3* and *SnRK2.6* were up-regulated in *smxl6/7/8* and down-regulated in overexpression lines of *SMXL6,7,8* (Figure S10). Together, these results suggest that SMXL6,7,8 proteins can directly bind to the promoters of drought-positive regulator/ABA signaling genes *SnRK2.3* and *SnRK2.6*, which repress their transcription.

### 3.5. SnRK2.2/2.3 Act Downstream of SMXL6,7,8 in Response to ABA

**Figure 6:**
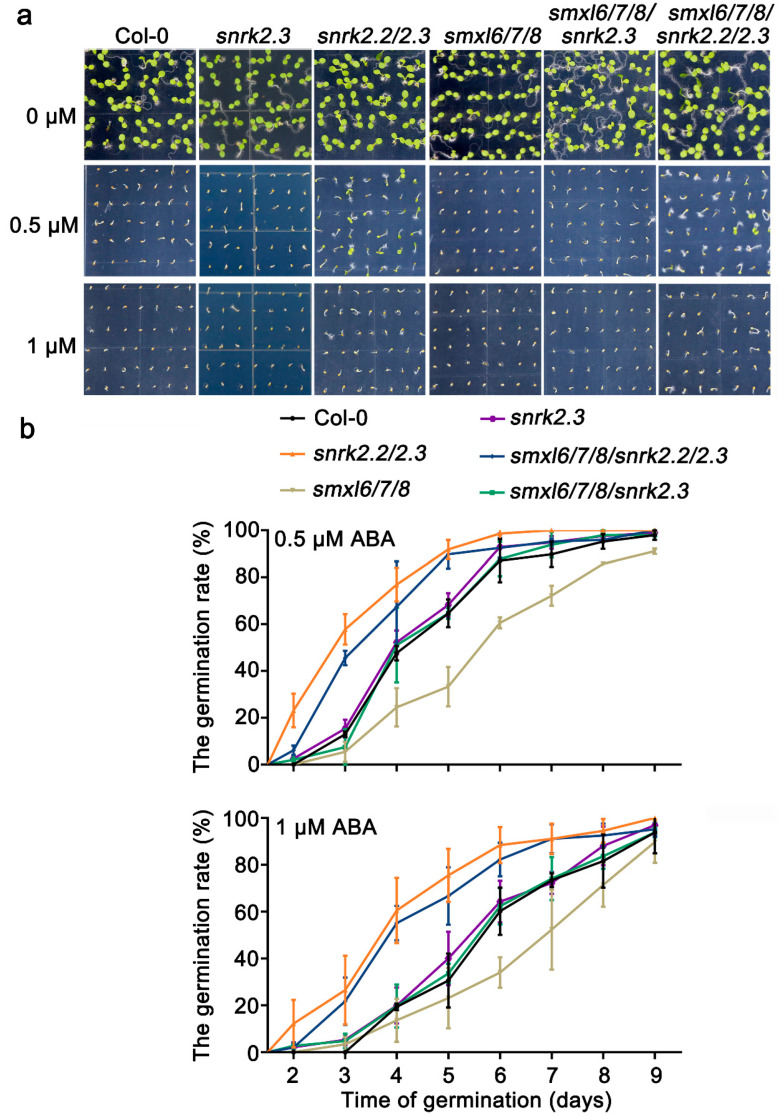
ABA hypersensitivity of *smxl6/7/8* requires functional *SnRK2.2/2.3*. (**a**) The seed germination phenotypes Col-0, *snrk2.3*, *snrk2.2/2.3*, *smxl6/7/8*, *smxl6/7/8snrk2.3*, and *smxl6/7/8/snrk2.2/2.3* mutants on plates at different ABA concentrations for 6 days after sowing. (**b**) The statistics of seed germination rate corresponding to the treatment under (**a**). Seed germination was recorded for the indicated times after sterilization on a medium supplemented with 0.5 μM and 1 μM ABA, respectively. The experiments described above were performed at least three times. For each biological replicate, we examined the seeds (more than 100) from the same batch three times as technical replicates.

Previous reports showed that SnRK2.2 and SnRK2.3 had functional redundancy and that the *snrk2.2/2.3* mutant was more insensitive to ABA than the wild type, *snrk2.2*, and *snrk2.3* single mutant during seed germination, while *smxl6/7/8* was more sensitive to ABA than the wild type [7,31]. Given that SMXL6,7,8 bound the *SnRK2.3* promoter and repressed its expression (Figure 5), we then asked whether SMXL6,7,8 were genetically related to SnRK2.3 to mediate ABA-induced repression of seed germination. Subsequently, we generated the *smxl6/7/8/snrk2.3* and *smxl6/7/8/snrk2.2/2.3* multiple mutants by crossing *smxl6/7/8* with *snrk2.2/2.3* and isolated the *snrk2.3* mutant by crossing *snrk2.2/2.3* with wild-type Col-0. The germination of Col-0, *snrk2.3*, *snrk2.2/2.3*, *smxl6/7/8*, *smxl6/7/8/snrk2.3*, and *smxl6/7/8/snrk2.2/2.3* was observed in response to ABA (Figure 6). In the absence of ABA, all genotypes exhibited no obvious differences in seed germination, but two concentrations of ABA treatments (0.5 μM and 1 μM) significantly inhibited seed germination (but to varying degrees). Consistent with previous findings, we also found that the germination rate of *smxl6/7/8* was much lower than that of the wild type, whereas that of *snrk2.2/2.3* was higher than both the wild type and *snrk2.3*. As expected, the *smxl6/7/8/snrk2.2/2.3* was also insensitive to ABA during seed germination compared to the wild type, which was similar to the *snrk2.2/2.3* seeds. In addition, the germination rate of *smxl6/7/8/snrk2.3* was higher than that of *smxl6/7/8*, which was similar to that of the *snrk2.3* seeds. That is, the mutation of SnRK2.2/2.3 repressed the effect of ABA on the seed germination in *smxl6/7/8*, indicating that SnRK2.2 and SnRK2.3 are functionally redundant and are located downstream of SMXL6,7,8 to participate in the ABA response.

## 4. Discussion

It has been shown that SLs have positive regulatory roles in plant adaptation to drought stress [17]. The *smxl6/7/8* triple mutant exhibited stronger drought tolerance with high ABA levels and upregulated drought-stress-inducible ABA-response genes [31]. In this study, the CUL4-based E3 ligase DWA1 physically interacted with SMXL6,7,8 in vivo and in vitro (Figure 1). DWA1 acts as a substrate receptor of CUL4-based E3 ligase that interacts with DWA2 and ABI5 to regulate ABA signaling by promoting the degradation of ABI5 [12]. We also assayed whether SMXL6,7,8 could directly interact with DWA2 and ABI5. However, the Y2H results showed that SMXL6,7,8 could interact with neither DWA2 nor ABI5 in yeast (Figure S11), indicating that SMXL6,7,8 specifically interacted with DWA1.

Ubiquitination is one of the most important post-translational protein modifications in eukaryotes and plays a key role in several cellular processes, including 26S proteasome-dependent protein degradation. In the CUL4-based E3 ligase complex, the substrate adapter DDB1 interacts with substrate receptors and binds to a large number of proteins to form the CUL4–DDB1 complex [46]. In turn, the substrate receptors interact with specific substrates that are targeted for degradation. The CUL4-based E3 ligase targets are key regulators of ABA responses. Another CUL4–DDB1 complex containing ABA-hypersensitive DCAF1 (ABD1) acts as a substrate receptor by directly binding to ABI5 and positively affecting its degradation [47]. DET1-DDB1-ASSOCIATED 1 (DDA1), as part of the CDDD (COP10-DET1-DDB1-DDA1) substrate adaptor module, targets the ABA receptor PYL8 for degradation [48]. As a DWD protein, RNA EXPORT FACTOR 1 (AtRAE1) may act as a substrate receptor of CUL4–DDB1 E3 ligase and participates in ABA signaling by regulating the degradation of the ABA receptor PYL9 [49]. In this study, DWA1 physically interacted with SMXL6,7,8, and the mutation of DWA1 partially delayed the degradation of SMXL6,7,8 both in vitro and in *OE-SMXL6,7,8/dwa1* transgenic plants (Figure 1, Figure 2 and Figure 3). Although we have preliminarily demonstrated that SMXL6,7,8 are partially dependent on DWA1 to be degraded, how the degradations of SMXL6,7,8 are triggered by DWA1 needs to be explored next. Previous studies have shown that the degradation of SMXL6,7,8 are dependent on MAX2 and that the GRKT motif is essential for the MAX2-mediated degradation of SMXL6,7,8 [23,24]. DWA1 and MAX2 show similar functions in the degradation of SMXL6,7,8 proteins and plant drought tolerance. Is there a possibility that DWA1 is a component of the SCF^MAX2^ complex to regulate the stability of SMXL6,7,8? Does mutation of the GRKT motif affect DWA1-mediated degradation of SMXL6,7,8? There needs to be further study using genetic and biochemical experiments, which will provide insight into the effect of DWA1 on the MAX2-mediated degradation of SMXL6,7,8.

Ubiquitin-conjugating enzymes and E3 ligases can negatively or positively participate in drought stress response. The endoplasmic reticulum-associated degradation (ERAD) component UBIQUITIN-CONJUGATING ENZYME 32 (UBC32) positively regulates drought tolerance in *Arabidopsis* by targeting the aquaporins PLASMA MEMBRANE INTRINSIC PROTEIN 2;1 (PIP2;1) and PIP2;2 for degradation [50]. The E3 ubiquitin ligases PUB22 and PUB23 negatively regulate drought tolerance by targeting the ABA receptor PYL9 for degradation in *Arabidopsis* [51]. In this study, *smxl6/7/8* and *smxl6/7/8/dwa1* exhibited the drought tolerance phenotype after 16 days of drought stress treatment compared with the wild type, and when the drought treatment time was extended, the drought tolerance of *smxl6/7/8/dwa1* was lower than that of *smxl6/7/8* after 18 days of drought stress treatment (Figure 4 and Figure S3). Together, these data suggest that SMXL6,7,8 are degraded by DWA1 through their interactions, thereby regulating drought tolerance in *Arabidopsis.*

A previous study reported that *dwa1* showed no difference in drought tolerance compared with the wild type [12]. In contrast, the *dwa1* mutant showed less tolerance after 16 days of drought stress treatment in this study (Figure 4, Figures S2c and S3). This phenotypic difference in the *dwa1* mutant in response to drought stress may be the difference in drought treatment time, and 16 days of drought treatment time was critical in our working condition and shorter than in Lee’s [12]. We also found that DWA1 partially promoted the degradation of SMXL6,7,8, and loss of DWA1 led to the accumulation of SMXL6,7,8 protein in planta (Figure 2 and Figure 3). In addition, the overexpression of *SMXL6,7,8* reduced drought tolerance compared with the wild type because of the negative regulatory function of SMXL6,7,8 (Figure S4). Thus, increased SMXL6,7,8 protein levels in *dwa1* may contribute to its drought-sensitive phenotype (Figure 4, Figures S2 and S3).

ABA-induced genes are also involved in regulating drought stress tolerance [8,44,52,53,54]. RELATED TO AP2 (RAP2) interacts with CYCLIN-DEPENDENT KINASE 8 (CDK8) and SnRK2.6 and positively modulates drought response in *Arabidopsis* [54]. In a previously published study, the results of a transcriptome analysis of *smxl6/7/8* after drought treatment showed that the drought treatment induced up-regulation of ABA-responsive genes in *smxl6/7/8*, indicating that SMXL6,7,8 may negatively regulate drought tolerance through an ABA-dependent manner [31]. The up-regulation of ABA-induced genes involved in drought stress and a high ABA content were concomitant with enhanced drought tolerance of the *smxl6/7/8* triple mutant [31].

In this study, SMXL6,7,8 could directly bind to the promoters of *SnRK2.3* and *SnRK2.6* (Figure 5 and Figure S8), which positively regulate ABA signaling and the drought stress response [8,55]. SMXL6,7,8, as transcription suppressors, inhibit the expression of downstream genes to respond to SL signaling by interacting with specific transcription factors [21,23]. SMXL6,7,8 also act as transcription factors to directly repress downstream gene expression, including that of themselves [30]. The overexpression of individual SMXL6, SMXL7, and SMXL8 inhibited the activity of LUC driven by the *SnRK2.3* and *SnRK2.6* promoters compared with the expression of GFP alone (Figure 5 and Figure S8). This suggests that the ABA/drought-response genes *SnRK2.3* and *SnRK2.6* are also transcriptionally regulated by SMXL6,7,8. The overexpression of *SnRK2.3* and *SnRK2.6* enhance drought tolerance [8]. The *snrk2.2/2.3/2.6* mutant significantly reduce the drought tolerance of *Arabidopsis*, while the overexpression of *TaSnRK2.3* in common wheat enhance the drought tolerance [10,11]. Meanwhile, AtPP2-B11, an F-box protein that is part of an SKP1/Cullin/F-box E3 ubiquitin ligase complex, has been reported to negatively regulate ABA signaling and the abiotic stress response by interacting with and targeting SnRK2.3 degradation [9]. It has been speculated that activation of SnRK2.3 may require other components in addition to the classic ABA signaling pathway [56,57]. The inactivation of SnRK2s is equally important to attenuate the ABA-induced drought response [58]. The relative expressions of *SnRK2.3* and *SnRK2.6* were up-regulated in the *smxl6/7/8* triple mutant (Figure S10). Genetic evidence suggests that *snrk2.3* and *snrk2.2/2.3* restored the ABA-sensitive phenotype of *smxl6/7/8* (Figure 6), indicating that SnRK2.2/2.3 were located downstream of SMXL6,7,8 and that ABA hypersensitivity of *smxl6/7/8* required functional *SnRK2.2/2.3* in seed germination. In addition, we will investigate whether SnRK2.6 is involved in the regulation of ABA response and drought tolerance downstream of SMXL6,7,8 in the future. It is well documented that the ATAACAA motif is essential for SMXL6 to directly bind to the promoter of *SMXL7* and repress its transcription [30]. However, in the present study, we did not observe that ATAACAA contributed to SMXL6,7,8 binding to the promoters of *SnRK2.3* and *SnRK2.6* (Figure 5 and Figure S8), which needs to be further explored.

## 5. Conclusions

**Figure 7:**
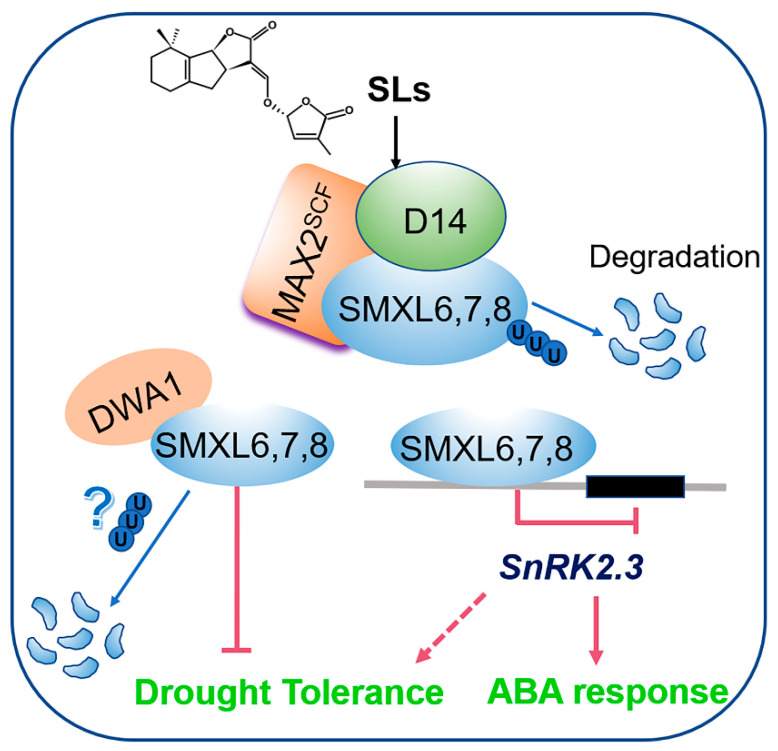
A schematic diagram showing that SMXL6,7,8 can negatively regulate drought tolerance and ABA response. On the one hand, DWA1 interacts with SMXL6,7,8 to partially promote the degradation of SMXL6,7,8, which repress the drought tolerance. On the other hand, SMXL6,7,8 act as transcription factors that bind to the promoter of the ABA-response gene *SnRK2.3*, repressing its transcription and negatively regulating ABA response.

Overall, a schematic diagram of how SMXL6,7,8 regulate drought tolerance was proposed (Figure 7). On the one hand, CUL4-based E3 ligase DWA1 functions upstream of SMXL6,7,8 to regulate plant drought stress responses by changing the stability of SMXL6,7,8 proteins. On the other hand, the SMXL6,7,8 bind to the promoter of *SnRK2.3* and repress its transcription, leading to an ABA response. Our study will prompt us to decipher whether homologous proteins of SMXL6,7,8 in rice, maize, and other crops can also play similar roles in response to drought stress tolerance and ABA, which will enhance a better understanding of the molecular mechanism of SL signaling in response to the drought stress tolerance of crops.

## Data Availability

The data presented in this study are available upon request from the corresponding author.

## Supplementary Materials

The following supporting information can be downloaded at: https://www.mdpi.com/article/10.3390/biom13091406/s1, Figure S1: Western blot analysis of SMXL6, SMXL7 and SMXL8 levels in *OE-SMXL6*, *OE-SMXL7* and *OE-SMXL8* lines, respectively; Figure S2: The *dwa1-1* mutants show drought sensitivity phenotype; Figure S3: The *smxl6/7/8* mutation represses the drought sensitivity phenotype of the *dwa1-1* mutant; Figure S4: The overexpression of *SMXL6,7,8* show drought sensitivity phenotypes; Figure S5: Toluidine blue staining of wild-type, *dwa1-1*, *dwa1-2*, *smxl6/7/8*, *smxl6/7/8/dwa1-1* and *smxl6/7/8/dwa1-2* mutant; Figure S6: GO-enriched analysis of targeted genes from SMXL6-bound ChIP-seq data; Figure S7: SMXL6,7,8 proteins bind to fragments ‘3-1-2-b’ of the *SnRK2.3* promoter *in vitro;* Figure S8: SMXL6,7,8 directly bind to the *SnRK2.6* promoter; Figure S9: SMXL6,7,8 do not bind to the promoter of *SnRK2.2* in yeast; Figure S10: Relative expression levels of *SnRK2.3* and *SnRK2.6* in Col-0, *smxl6/7/8* triple mutant and overexpression of *SMXL6,7,8*; Figure S11: SMXL6,7,8 do not interact with DWA2 and ABI5 in yeast; Table S1: Summary of primers used in this study.
