# Supplementary material for: SUPPRESSOR OF MAX2 LIKE 6, 7, and 8 Interact with DDB1 BINDING WD REPEAT DOMAIN HYPERSENSITIVE TO ABA DEFICIENT 1 to Regulate the Drought Tolerance and Target SUCROSE NONFERMENTING 1 RELATED PROTEIN KINASE 2.3 to Abscisic Acid Response in Arabidopsis
Source: Biomolecules. 2023 Sep 18;13(9):1406. doi: 10.3390/biom13091406 (PMC10526831; doi:10.3390/biom13091406)
Supplement: Supplementary file 1 [file biomolecules-13-01406-s001.zip › Supplemental materials.pdf]

Supplemental materials:

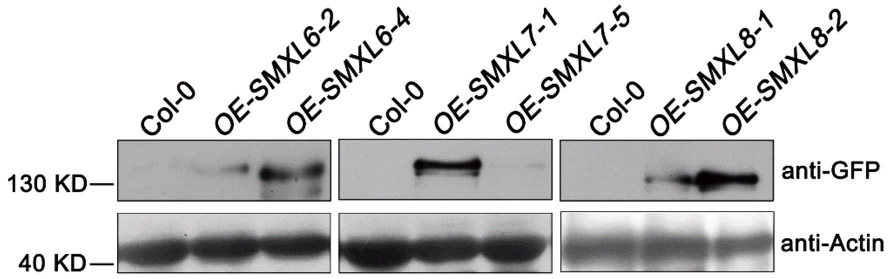

Figure S1 Western blot analysis of SMXL6, SMXL7 and SMXL8 levels in *OE-SMXL6*, *OE-SMXL7* and *OE-SMXL8* lines, respectively.

7-day-old seedlings of Col-0, *OE-SMXL6*, *OE-SMXL7* and *OE-SMXL8* lines grown on ½ MS were extracted in a buffer (1 M Tris-MES, pH 8.0, 2 M sucrose, 1 mM $MgCl_2$, 0.5 M EDTA, 2 M DTT and 0.1 M PMSF). Equal amounts of samples were separated by 8% SDS-PAGE gels and analyzed by immunoblotting with an anti-GFP antibody. Anti-Actin antibody was used as a normalization control.

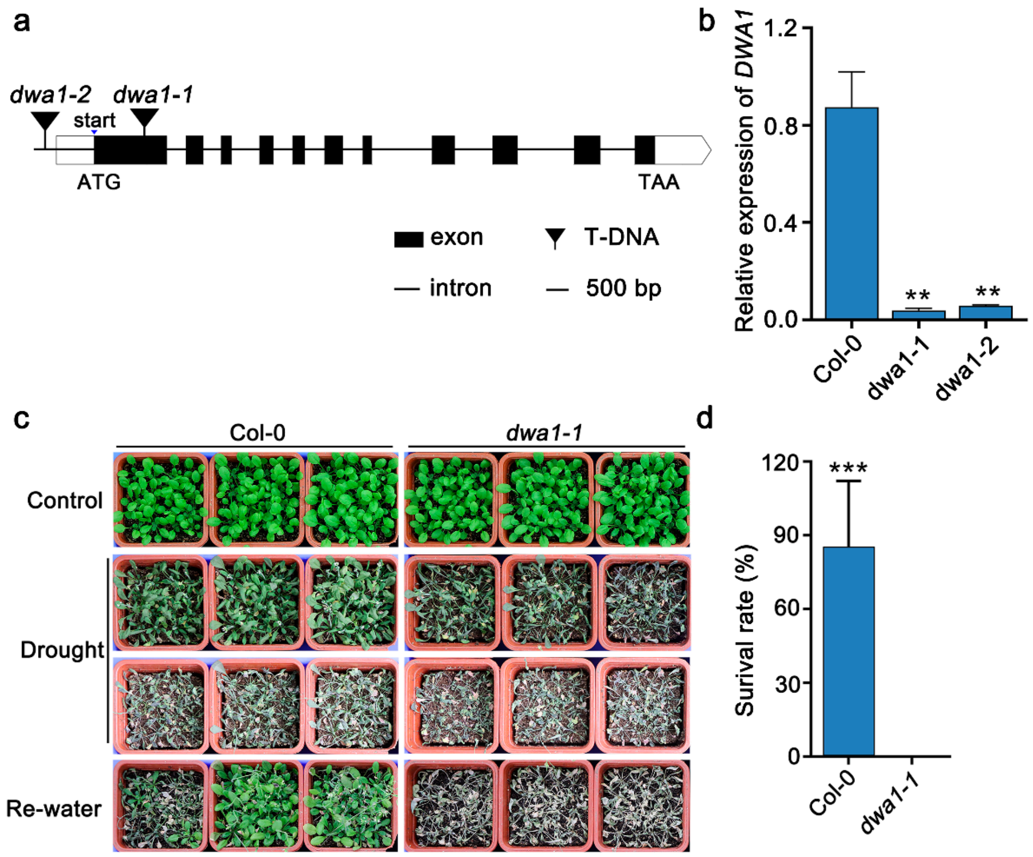

Figure S2 The *dwa1-1* mutants show drought sensitivity phenotype.

a T-DNA insertion positions for *dwa1-1* and *dwa1-2*. Black boxes indicate exons, straight lines indicate intron and dark triangles indicate the positions of T-DNAs. **b** The relative expression of *DWA1* in *dwa1-1* and *dwa1-2* mutants. *ACTIN2* was used as a reference gene. Data were means of three biological replicates ± SD. **c** Drought tolerance assay of wild-type (Col-0) and *dwa1-1* mutant. 3 weeks plants were treated with drought stress for 16 days and then re-watered for 3 days. **d** Survival rates of wild-type and *dwa1-1* after 3 days recovery. Values are means ± SD ($n = 3$). Asterisks indicate a significant difference (**$P < 0.01$, ***$P < 0.001$) as determined by Student's t-test.

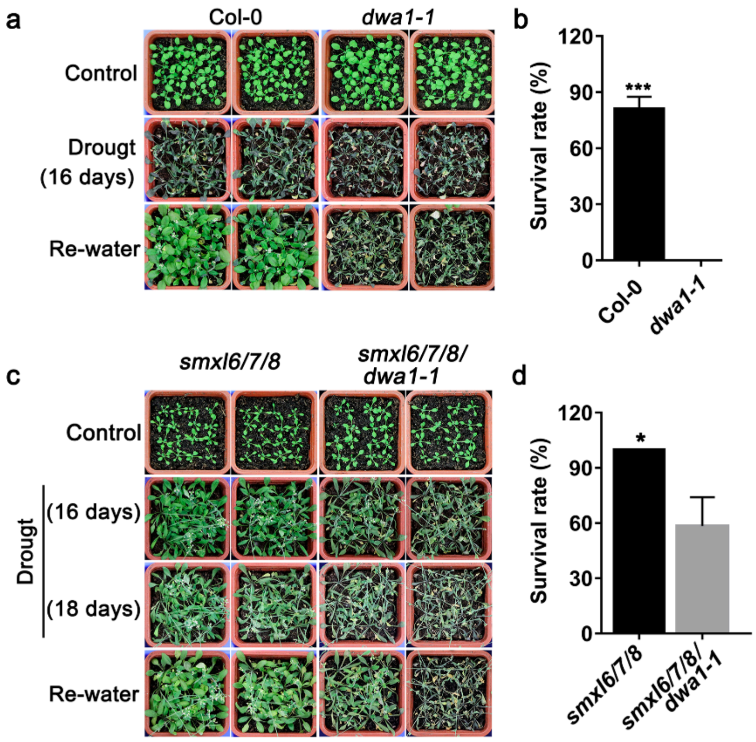

Figure S3 The *smxl6/7/8* mutation represses the drought sensitivity phenotype of the *dwa1-1* mutant. **a, c** Drought tolerance assay of wild-type and *dwa1-1* (**a**), *smxl6/7/8* and *smxl6/7/8/dwa1-1* (**c**). 3 weeks plants were treated with drought stress for 16 days (**a**) or 18 days (**c**) and then re-watered for 3 days. **b, d** Survival rates of wild-type and *dwa1-1* (**b**), *smxl6/7/8* and *smxl6/7/8/dwa1-1* (**d**) after 3 days recovery. Values are means ± SD (n = 3). Asterisks indicate a significant difference (*$P < 0.05$, ***$P < 0.001$) as determined by Student's t-test.

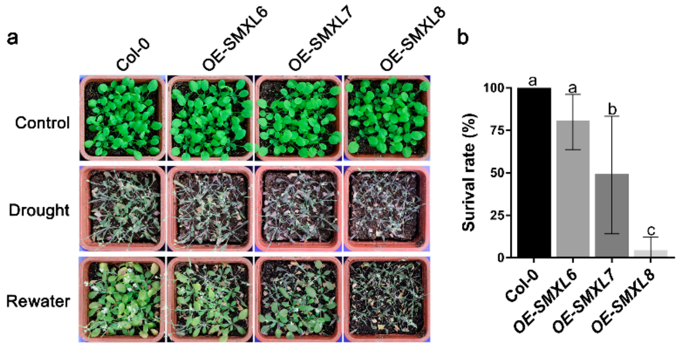

Figure S4 The overexpression of *SMXL6,7,8* show drought sensitivity phenotypes.

a Drought tolerance assay of wild-type, *OE-SMXL6*, *OE-SMXL7* and *OE-SMXL8*. Three weeks plants were treated with drought stress for 16 days and then re-watered for 3 days. **b** Survival rates of wild-type, *OE-SMXL6*, *OE-SMXL7* and *OE-SMXL8* after 3 days recovery. Values are means ± SD (n = 3). Different letters represent statistical significance at $P < 0.05$ (by Duncan's multiple range test).

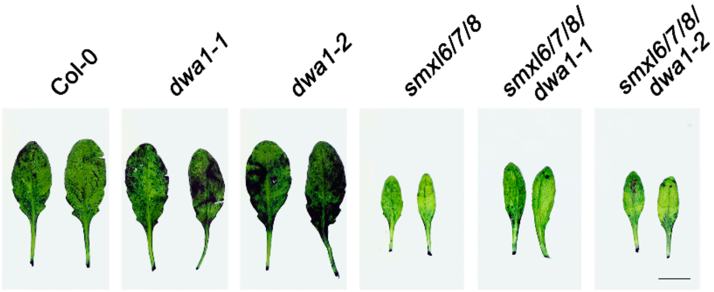

Figure S5 Toluidine blue staining of wild-type, *dwa1-1*, *dwa1-2*, *smxl6/7/8*, *smxl6/7/8/dwa1-1* and *smxl6/7/8/dwa1-2* mutant.

The fifth leaves from different lines of 4-week-old plants were stained with toluidine blue and washed with water and photographed. Bars, 1 cm. The experiments were repeated three times with similar results.

a

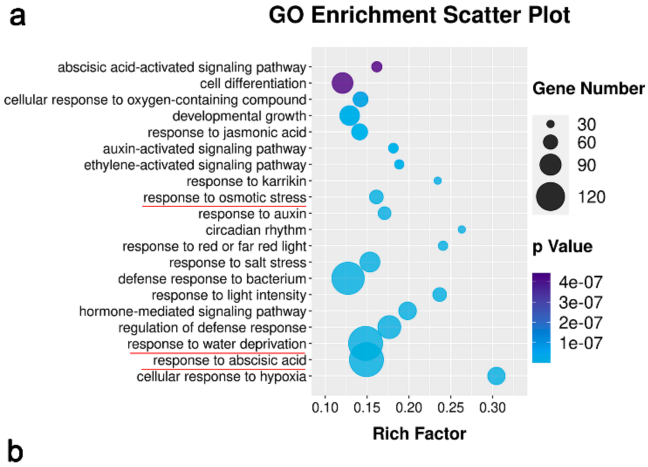

b

Gene ID	Gene name	Gene ID	Gene name
AT2G36270	*ABI5*	AT2G38310	*PYL4*
AT1G13740	*AFP2*	AT5G05440	*PYL5*
AT1G69260	*AFP1*	AT1G01360	*PYL9*
AT3G48360	*BT2*	AT5G66880	*SnRK2.3*

Figure S6 GO-enriched analysis of targeted genes from SMXL6-bound ChIP-seq data.

a Deep clustering analysis of SMXL6 ChIP-seq data. GO enrichment analysis showed that 729 genes targeted by SMXL6-HA (in two replicates) were partially involved in ‘response to abscisic acid’, ‘response to water deprivation’, ‘response to osmotic stress’ and other hormone response pathways. **b** Candidate target genes associated with ‘response to abscisic acid’ from ChIP-seq data.

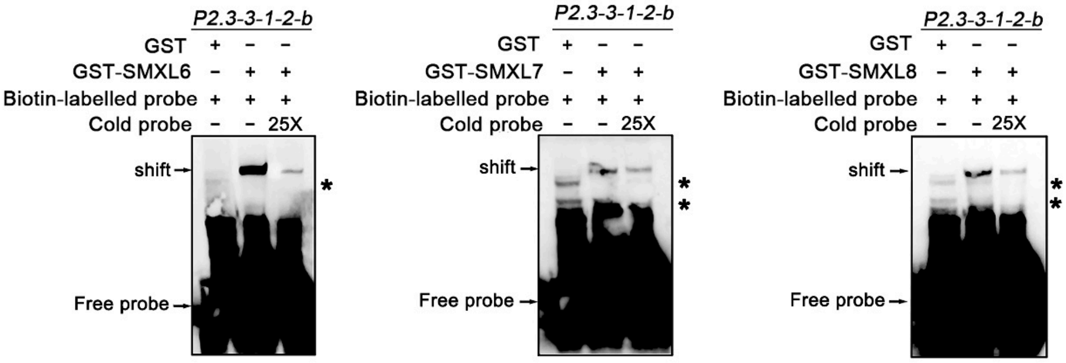

Figure S7 SMXL6,7,8 proteins bind to fragments '3-1-2-b' of the *SnRK2.3* promoter *in vitro*. For the competition, 25x excess unlabelled probes (cold probes) were mixed with biotin-labelled probes. GST, glutathione-S-transferase. Asterisks indicate nonspecific binding.

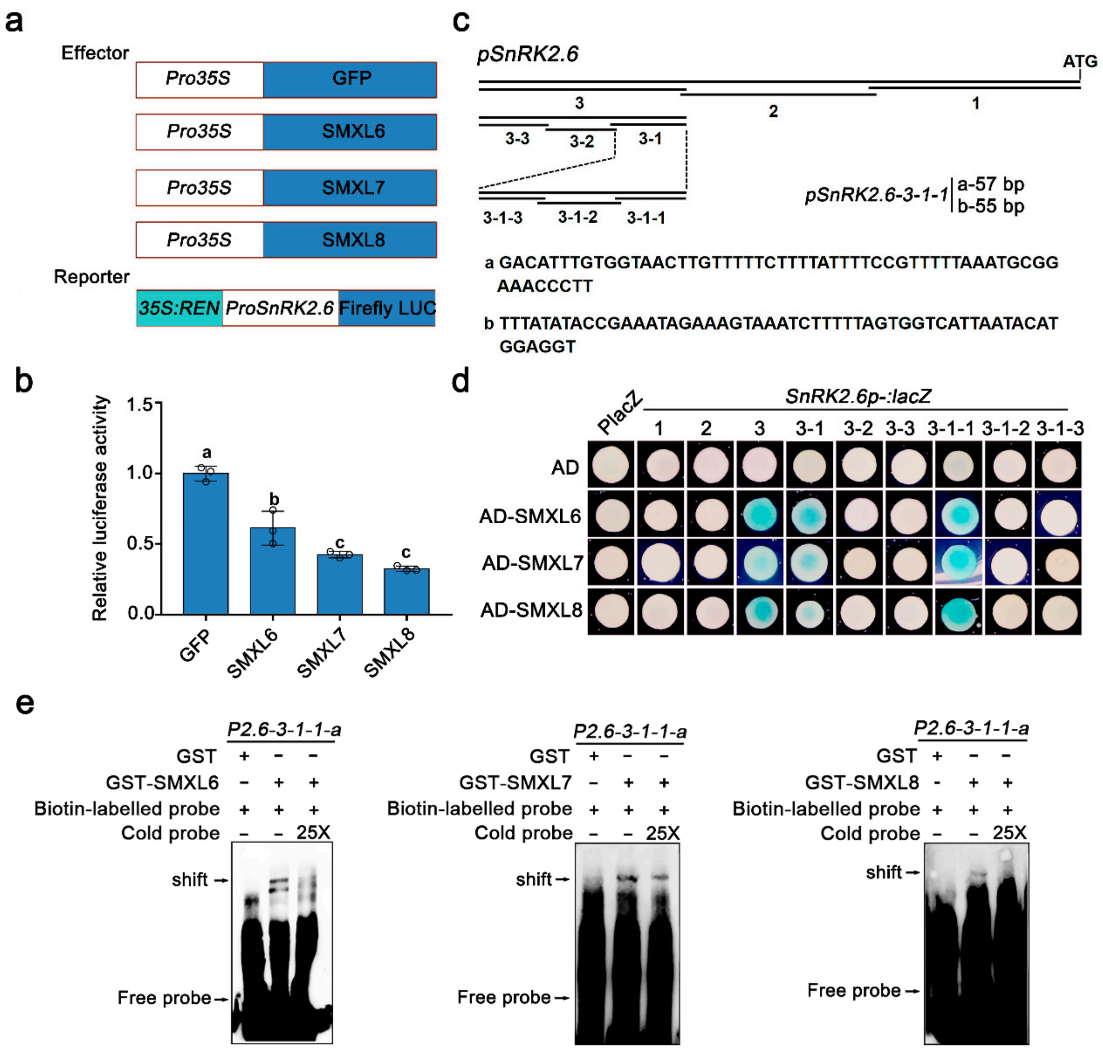

Figure S8 SMXL6,7,8 directly bind to the *SnRK2.6* promoter.

a Schematic diagram of dual-luciferase assay constructs. The promoter of *SnRK2.6* was fused to the *REN* gene controlled by a *35S* promoter and *LUC* gene as a reporter, and the effector constructs contained a *GFP*, *SMXL6*, *SMXL7* or *SMXL8* gene driven by the CaMV 35S promoter. **b** Transcriptional activities of SMXL6,7,8 on the *SnRK2.6* promoter in *Arabidopsis* protoplasts. The experiment was independently repeated three times with three biological replicates in each experiment, and similar results were obtained. Data were means of three biological replicates ± SD. Different letters represent statistical significance at $P < 0.05$ (by Duncan's multiple range test). **c** Truncated regions of the *SnRK2.6* promoter and synthesized fragments of EMSA probes. **d** Yeast one-hybrid assays showing that SMXL6,7,8 displayed strong binding to the fragment '3-1-1' of the *SnRK2.6* promoter. An empty vector expressing the AD domain alone was used as the negative control. **e** EMSAs showing that the SMXL6 and SMXL8 proteins specifically bind to fragments '3-1-1-a' of the *SnRK2.6* promoter *in vitro*. For the competition, 25x excess

unlabelled probes (cold probes) were mixed with biotin-labelled probes. GST, glutathione-S-transferase.

Data represent three independent experiments.

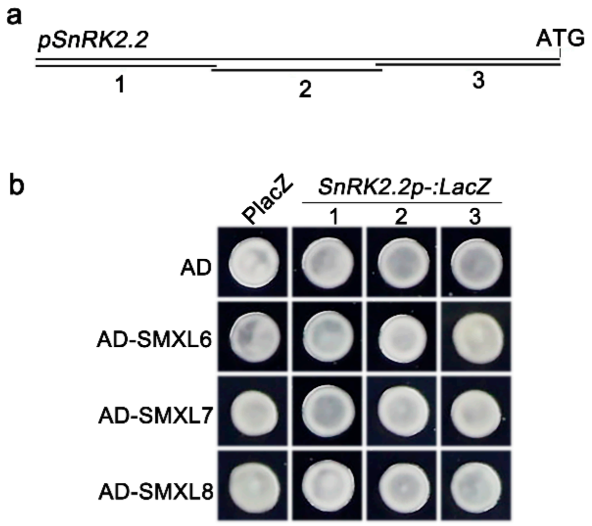

Figure S9 SMXL6,7,8 do not bind to the promoter of *SnRK2.2* in yeast.

a Different truncated regions of the *SnRK2.2* promoter. **b** Yeast one-hybrid assays showing that SMXL6,7,8 do not bind to the *SnRK2.2* promoter. An empty vector expressing the AD domain alone was used as the negative control.

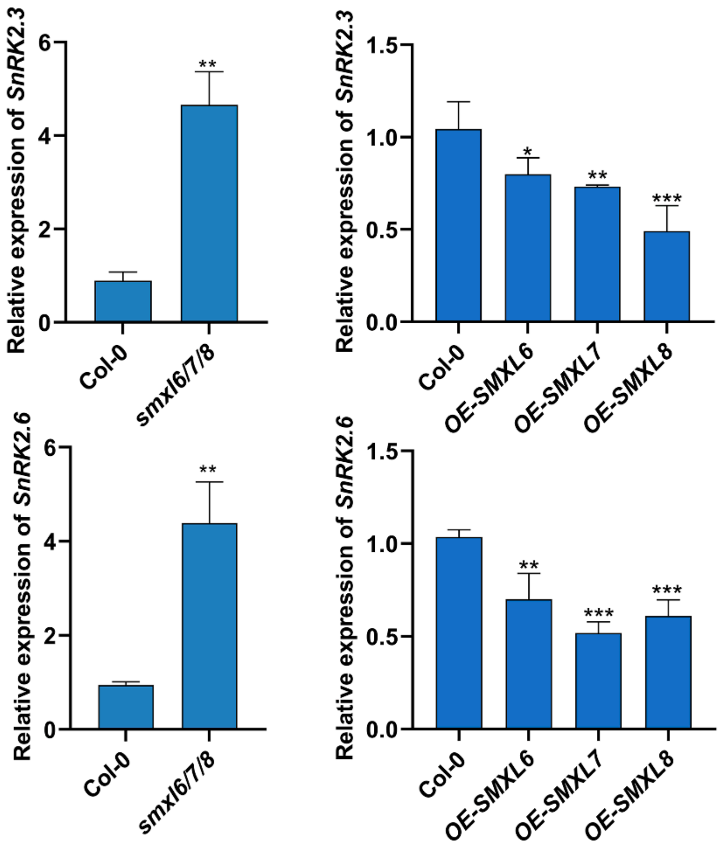

Figure S10 Relative expression levels of *SnRK2.3* and *SnRK2.6* in Col-0, *smxl6/7/8* triple mutant and overexpression of *SMXL6,7,8*.

SnRK2.3/2.6: *SUCROSE NONFERMENTING 1 (SNF1)-RELATED PROTEIN KINASE 2.3/2.6*. The Arabidopsis *ACTIN2* gene was used as the internal control. All experiments were set up with three biological replicates and three technical replicates. Values are means ± SD. Asterisks indicate a significant difference (*$P < 0.05$, **$P < 0.01$, ***$P < 0.001$) as determined by Student's *t*-test.

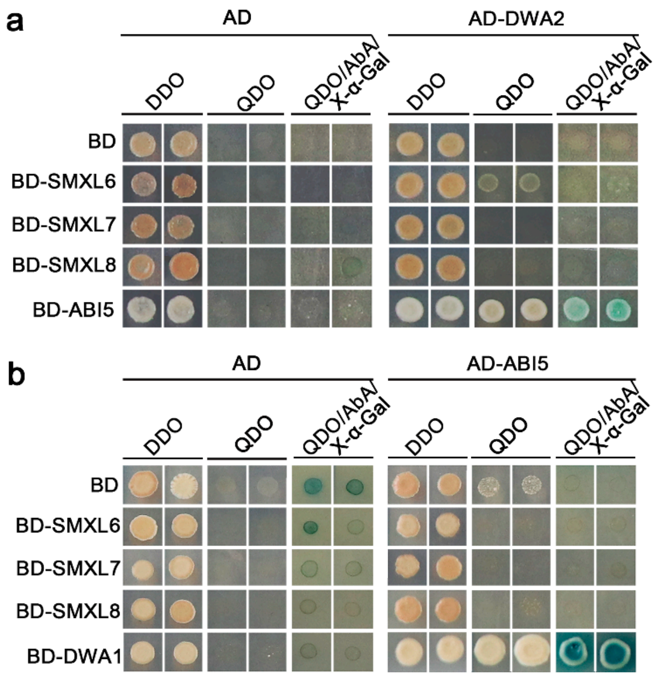

Figure S11 SMXL6,7,8 do not interact with DWA2 and ABI5 in yeast.

a SMXL6,7,8 do not interact with DWA2 in yeast. **b** SMXL6,7,8 do not interact with ABI5 in yeast. The full-length SMXL6,7,8 proteins and DWA1 and ABI5 proteins were fused to the GAL4 binding domain (BD), while the DWA2 and ABI5 proteins were fused to the GAL4 activation domain (AD). Constructs were co-transformed into Y2H Gold and spotted onto DDO, QDO and QDO/ X-α-Gal/AbA. DDO: SD/-Leu/-Trp; QDO: SD/–Ade/–His/–Leu/–Trp; X-α-Gal: 5-Bromo-4-chloro-3-indoxyl-α-D-galactopyranoside; AbA: Aureobasidin A (a cyclic, depsipeptide antifungal antibiotic).
